# Arterial Pulsatility Augments Microcirculatory Perfusion and Maintains the Endothelial Integrity during Extracorporeal Membrane Oxygenation via hsa_circ_0007367 Upregulation in a Canine Model with Cardiac Arrest

**DOI:** 10.1155/2022/1630918

**Published:** 2022-02-18

**Authors:** Guanhua Li, Shenyu Zhu, Jianfeng Zeng, Zhexuan Yu, Fanji Meng, Zhixian Tang, Ping Zhu

**Affiliations:** ^1^Department of Cardiovascular Surgery, Guangdong Cardiovascular Institute, Guangdong Provincial Key Laboratory of South China Structural Heart Disease, Guangdong Provincial People's Hospital, Guangdong Academy of Medical Sciences, Guangzhou 510080, China; ^2^Department of Cardiovascular Surgery, Sun Yat-sen Memorial Hospital, Sun Yat-sen University, Guangzhou 510120, China; ^3^Department of Cardiothoracic Surgery, The First Affiliated Hospital of Gannan Medical University, Ganzhou 341000, China; ^4^Ganzhou Key Lab of Brain Injury & Brain Protection, Ganzhou 341000, China; ^5^Department of Anesthesiology, Sun Yat-sen Memorial Hospital, Sun Yat-sen University, Guangzhou 510120, China; ^6^Zhejiang Chinese Medical University, Hangzhou 310053, China; ^7^Department of Cardiology, The Fourth Affiliated Hospital of Harbin Medical University, Harbin 150001, China

## Abstract

**Background:**

The impairment of microcirculation is associated with the unfavorable outcome for extracorporeal membrane oxygenation (ECMO) patients. Studies revealed that pulsatile modification improves hemodynamics and attenuates inflammation during ECMO support. However, whether flow pattern impacts microcirculation and endothelial integrity is rarely documented. The objective of this work was to explore how pulsatility affects microcirculation during ECMO.

**Methods:**

Canine animal models with cardiac arrest were supported by ECMO, with the i-Cor system used to generate nonpulsatile or pulsatile flow. The sublingual microcirculation parameters were examined using the CytoCam microscope system. The expression of hsa_circ_0007367, a circular RNA, was measured during ECMO support. In vitro validation was performed in pulmonary vascular endothelial cells (PMVECs) exposed to pulsatile or nonpulsatile flow, and the expressions of hsa_circ_0007367, endothelial tight junction markers, endothelial adhesive molecules, endothelial nitric oxide synthases (eNOS), and NF-*κ*B signaling activity were analyzed.

**Results:**

The pulsatile modification of ECMO enhanced microcirculatory perfusion, attenuated pulmonary inflammation, and stabilized endothelial integrity in animal models; meanwhile, the expression of hsa_circ_0007367 was significantly upregulated both in animals and PMVECs exposed to pulsatile flow. In particular, upregulation of hsa_circ_0007367 stabilized the expressions of endothelial tight junction markers zonula occludens- (ZO-) 1 and occludin, followed by modulating the endothelial nitric oxide synthases (eNOS) activity and inhibiting the NF-*κ*B signaling pathway.

**Conclusion:**

The modification of pulsatility contributes to microcirculatory perfusion and endothelial integrity during ECMO. The expression of hsa_circ_0007367 plays a pivotal role in this protective mechanism.

## 1. Introduction

Extracorporeal membrane oxygenation (ECMO) is an important device in critical care medicine, especially during the COVID-19 pandemic [1]. Indeed, ECMO is so effective that the American Heart Association (AHA) guidelines on cardiopulmonary resuscitation (CPR) highly recommend ECMO in critical patients who are refractory to traditional means of life support [2]. ECMO replaces the cardiorespiratory function and reverses the macrocirculatory hemodynamics speedily; however, whether ECMO overturns the compromised microcirculation after the support is uncertain. Microcirculation impairment is closely associated with the undesirable high mortality for ECMO patients [3].

Microcirculation malperfusion exaggerates systemic inflammation, which subsequently damages the endothelial barrier, leading to capillary leakage and vascular permeability breakdown [4]. The ECMO centrifugal pump typically generates flow in a nonpulsatile manner and its main disadvantage includes the inadequacy of microcirculatory perfusion [5]. Few ECMO pumps produce pulsatile flow, while the i-Cor ECMO device (Xenios AG, Heilbronn, Germany), firstly applied in Germany [6], delivers pulsatile flow which is triggered by the electrocardiogram. We previously demonstrated that pulsatile modification attenuates systemic inflammatory responses [7] and protects the glycocalyx by maintaining proper pulsatile shear stress [8] when introducing the i-Cor system to ECMO animal models, but the potential mechanism remains poorly understood.

Recent studies have suggested a significant role of circular RNAs (circRNAs) in regulating the pathogenesis in various cardiovascular diseases [9]. To investigate the underlying epigenetic mechanism of pulsatility, we used bioinformatics previously to explore differentially expressed genes and noncoding RNAs between pulsatile or nonpulsatile flow in patients with ventricular assisting devices [10]. The circular RNA (circRNA) hsa_circ_0007367, a putative circRNA originating from the ubiquitin-associated protein 2 (UBAP2) mRNA, was initially identified to be tightly interrelated to the pathophysiology of pulsatility. In the current study, we aimed to investigate how hsa_circ_0007367 (circUBAP2) interacts with pulsatility and improves microcirculatory homeostasis during ECMO.

## 2. Materials and Methods

### 2.1. Animals and ECMO Instrumentation

Ten beagles purchased from the Laboratory Animal Center of the Southern Medical University were included in our work. Animals were randomized and divided into two groups: the nonpulsatile group (control group) and the pulsatile group (intervention group). Animals were anesthetized and managed as described previously [7, 8]. In brief, anesthesia was maintained using fentanyl (150 *μ*g/kg) and 2.5% sevoflurane. Animals were then tracheotomized and ventilated using an animal mechanical ventilator (HX-300S, Taimeng Inc., Chengdu, China). Animal models with cardiac arrest were created using a 4 V alternating current to induce ventricular fibrillation. After unfractionated heparin bolus, we cannulated the right jugular vein and advanced into the right atrium with a 10 Fr cannula (Medtronic Inc., Minneapolis, MN, USA). The ECMO inflow was established by an 8 Fr cannula (Medtronic Inc., Minneapolis, MN, USA) which was cannulated to the right common carotid artery.

The circuit was connected to the i-Cor system (Xenios AG, Heilbronn, Germany) with a diagonal pump and a membrane oxygenator (Medos Medizintechnik AG, Stolberg, Germany). The i-Cor system enabled simply switching flow modes (nonpulsatile or pulsatile) and delivered pulsatile flow at an equivalent rate (1 : 1). Venous-arterial (V-A) ECMO was implemented at 130 mL/kg/min and was maintained with an activated clotting time (ACT) of approximately 200 seconds. Macrocirculatory hemodynamics, blood gas analyses, and microcirculatory parameters were collected at baseline (*T*_0_), 1 hour (*T*_1_), 2 hours (*T*_2_), 4 hours (*T*_3_), and 6 hours (*T*_4_). After 6 hours of ECMO support, lung tissues were collected and stored for molecular evaluations.

### 2.2. Microcirculation Assessment

The CytoCam microscope system (Braedius Medical, Huizen, The Netherlands) was applied to assess the microcirculation of the sublingual area. Incident dark field (IDF) images of sublingual microcirculation were collected thrice. Video clips with acceptable quality were recorded and analyzed by only one investigator through the AVA 3.0 software (University of Amsterdam, the Netherlands). Only vessels less than 20 *μ*m with blood flow were counted. Microvascular flow index (MFI) and perfused vessel density (PVD) were calculated and were reported according to the second consensus on the assessment of sublingual microcirculation in critically ill patients from the European Society of Intensive Care Medicine [11].

### 2.3. Pulmonary Microvascular Endothelial Cell (PMVEC) Culture

10 Sprague Dawley (SD) rats were obtained from the Laboratory Animal Center of the Southern Medical University. The SD rats were euthanized, and the lung tissues were obtained after removing the pleura and large vessels. Tissues were then stored in culture flasks and were cultivated at room temperature for 4 days. PMVECs were isolated subsequently, with mediums changed at an interval of two days. PMVECs were cultured according to the proven techniques in our laboratory, as described previously [8].

### 2.4. In Vitro Pulsatile Experiments

In vitro pulsatile experiments were performed using the Flexcell apparatus (Flexcell™ Inc., McKeesport, PA, USA), which produced pulsatility to PMVECs. Seeded PMVECs (1 × 10^5^ cells/well) on 6-well Flexcell plates were deprived of fetal bovine protein and were exposed to continuous flow (0 dyne/cm^2^) or pulsatile flow (5 dyne/cm^2^), with frequency and flow rate set at 1 Hz and 2 mL/min, respectively. PMVECs were cultured and treated with pulsatile or nonpulsatile flow under different conditions for 6 hours, as we described previously [8].

### 2.5. Quantitative RT-PCR

Total RNA from tissues and PMVECs was extracted with the Trizol reagent (Thermo Fisher Scientific, MA, USA). The extracted RNA was subjected to reverse transcription, and the complementary DNA was made using the Oligo (dT) primers. Quantitative PCR samples were prepared by mixing complementary DNAs, power-SYBR Mix (Yeason Biotech Co., Shanghai, China), and specific primers (Table 1). Real-time PCR was carried out thrice for each experiment using the LightCycler 480 (Roche, Basel, Switzerland). Gene expression levels were normalized to GADPH.

### 2.6. Western Blotting

PMVECs were lysed with RIPA lysis buffer with protease inhibitors. Protein concentrations were examined, and cell lysates were subjected to SDS-PAGE. After transferring to the nitrocellulose membranes and blocking with 5% skimmed milk, primary antibodies against zonula occludens- (ZO-) 1, occludin, VCAM-1, ICAM-1, NF-*κ*B, endothelial nitric oxide synthases (eNOS), or GADPH were added to incubate overnight at 4°C. After washing properly, the secondary antibodies were added to the membrane to incubate for 2 hours at room temperature. Using GADPH as controls, the expressions of proteins were visualized with the ECL system.

### 2.7. Immunofluorescence

PMVECs were fixed with 4% paraformaldehyde for half an hour, followed by permeabilization with PBS containing 0.01% Triton X-100. Samples were incubated with PBS containing 3% bovine serum albumin for 1 hour and were then incubated overnight with primary antibodies against ZO-1 and occludin. PMVECs were washed three times with PBS and were cultivated with secondary antibodies. With the nuclei stained with DAPI, the mounted slides were examined by a fluorescence microscope.

### 2.8. Statistics

Statistical analyses were performed with IBM SPSS Statistics version 21.0 software (SPSS Inc., Chicago, IL, USA). Data were checked for distribution and homogeneity of variance before analyses. Continuous data were expressed as mean ± standard deviation (SD) if normally distributed; otherwise, the median (interquartile range) was used. The repeated measures of analysis of variance (ANOVA) was used to compare between-group differences at different time points in normally distributed variables, whereas the Mann–Whitney *U* test and Friedman test were applied to compare between-group and within-group variations in nonnormally distributed data, respectively. A *p* value below 0.05 was assumed to be statistically significant.

## 3. Results

All animals survived during the experiment. Most circuits went smoothly, with blood flow above 80 mL/kg/min. The circuit blood flow, ACT, and the time from shock to ECMO establishment did not differ between these two groups. The time from cardiac arrest to ECMO initiation was 31.2 ± 9.7 minutes in the nonpulsatile group versus 28.7 ± 11.4 minutes in the pulsatile group. As shown in Table 2, ECMO improved macrocirculation and hemodynamics, while the hemoglobin level decreased during ECMO support. However, there were no significant differences observed between these two groups for macrocirculatory and blood gas parameters, including mean arterial pressure (MAP), pH, arterial partial pressure of oxygen (PaO_2_), hemoglobin (Hb), and arterial oxygen saturation (SaO_2_). The mean dosage for noradrenaline showed no remarkable between-group differences.

### 3.1. Pulsatility Improves the Microcirculatory Hemodynamics during ECMO

**Figure 1:**
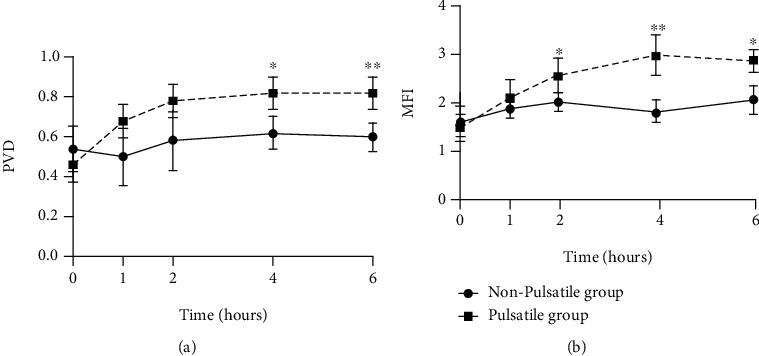
Pulsatility improves the microcirculatory parameters in on-ECMO animal models with cardiac arrest. (a) Animals were exposed to nonpulsatile or pulsatile circuits, and PVD were measured at different time points; (b) animals were exposed to nonpulsatile or pulsatile circuits, and MFI were measured at different time points. ^∗^Significantly different between groups, *p* < 0.05; ^∗∗^significantly different between groups, *p* < 0.01. Abbreviations: ECMO: extracorporeal membrane oxygenation; PVD: perfused vessel density; MFI: microvascular flow index.

Using the CytoCam microscope system, the PVD and MFI were measured at different time points, as shown in Figure 1. The pulsatile group had higher PVD values after 4 hours of ECMO support than did the nonpulsatile group (Figure 1(a)). Meanwhile, MFI was significantly higher in the pulsatile group as compared to the nonpulsatile group after 2 hours of ECMO support (Figure 1(b)).

**Figure 2:**
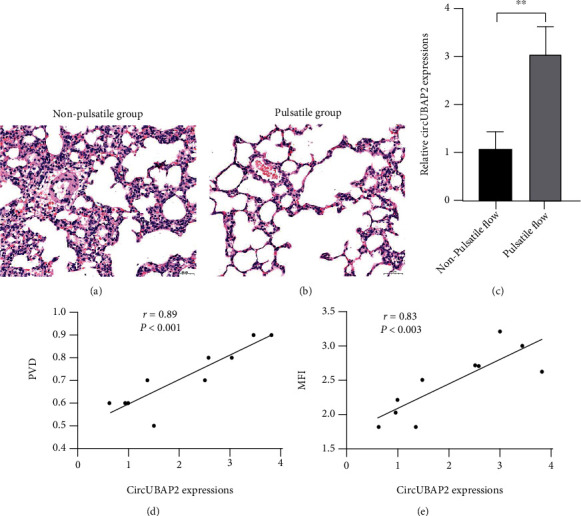
Pulsatility attenuates lung injury and upregulates the expression of hsa_circ_0007367 (circUBAP2), which is a microcirculation-related circRNA. (a) Representative images of lung tissues with hematoxylin and eosin staining in nonpulsatile group animals after 6 hours of ECMO; (b) representative images of lung tissues with hematoxylin and eosin staining in pulsatile group animals after 6 hours of ECMO; (c) comparison of the circUBAP2 expressions in lung tissues between these two groups; (d) correlation analysis between the expression of circUBAP2 and the PVD value; (e) correlation analysis between the expression of circUBAP2 and the MFI value. ^∗∗^Significantly different between groups, *p* < 0.01. Abbreviations: PVD: perfused vessel density; MFI: microvascular flow index.

As shown in Figures 2(a) and 2(b), lung tissues of the pulsatile group demonstrated decreased pulmonary injury after ECMO support as compared to the nonpulsatile group, presenting as attenuated infiltration of inflammatory cells and less capillary leakage. When comparing the expressions of hsa_circ_0007367 in lung tissues after 6 hours of ECMO support, the pulsatile group animals had significantly higher hsa_circ_0007367 levels (Figure 2(c)); moreover, positive correlations were observed between the expressions of hsa_circ_0007367 and the microcirculatory parameters (Figures 2(d) and 2(e)), inferring that hsa_circ_0007367 is closely related to microcirculatory perfusion.

### 3.2. Pulsatility Protects the Endothelial Integrity and Preserves the Expressions of ZO-1 and Occludin

**Figure 3:**
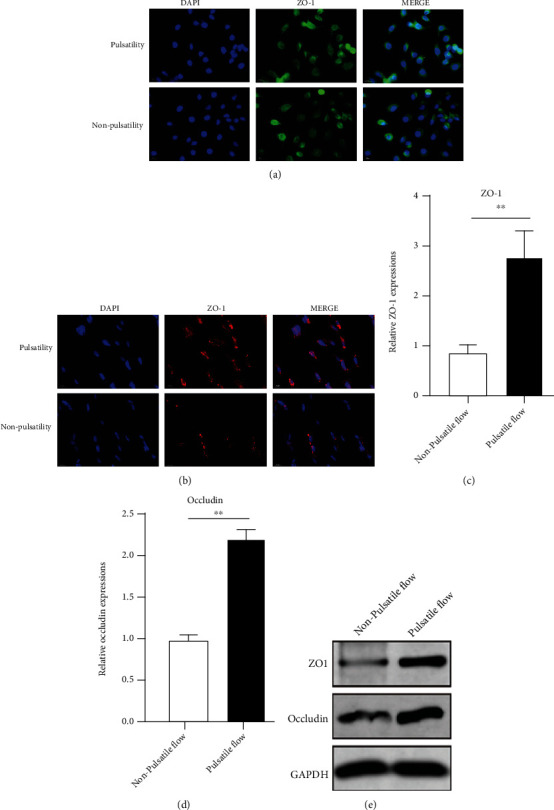
Pulsatility maintains the expressions of ZO-1 and occludin. (a) Immunofluorescent analysis of the ZO-1 distribution in PMVECs exposed to nonpulsatile or pulsatile flow for 6 hours; (b) immunofluorescent analysis of the occludin distribution in PMVECs exposed to nonpulsatile or pulsatile flow for 6 hours; (c) the expression of ZO-1 in PMVECs exposed to nonpulsatile or pulsatile flow for 6 hours using qPCR; (d) the expression of occludin in PMVECs exposed to nonpulsatile or pulsatile flow for 6 hours using qPCR; (e) the expressions of ZO-1 and occludin in PMVECs exposed to nonpulsatile or pulsatile flow for 6 hours using western blot assay. ^∗∗^Significantly different between groups, *p* < 0.01. Abbreviations: ZO-1: zonula occludens-1; DAPI: 4′,6-diamidino-2-phenylindole; PMVEC: pulmonary microvascular endothelial cell.

To investigate how ECMO flow pattern affects the endothelial integrity, cultured PMVECs were exposed to continuous flow (0 dyne/cm^2^) or pulsatile flow (5 dyne/cm^2^) for 6 hours, and the expressions of tight junction biomarkers were measured, including ZO-1 and occludin. As shown by the immunofluorescent analyses, the distribution of ZO-1 and occludin was disrupted in PMVECs exposed to nonpulsatile flow, while PMVECs treated with pulsatile flow showed more normal distribution of the endothelial tight junction proteins (Figures 3(a) and 3(b)). Moreover, qPCR and western blot demonstrated that the expressions of ZO-1 and occludin were upregulated in PMVECs of the pulsatile group as compared to the nonpulsatile group (Figures 3(c)–3(e)).

### 3.3. hsa_circ_0007367 (UBAP2) Preserves the Endothelial Barrier in PMVECs

**Figure 4:**
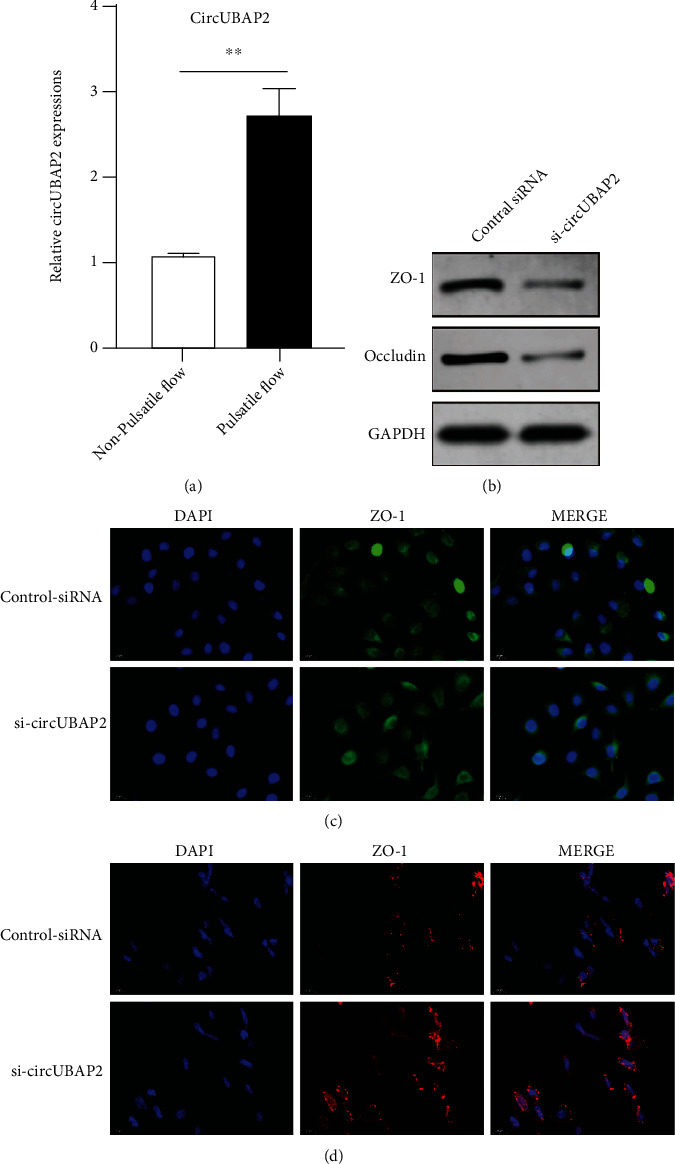
hsa_circ_0007367 (UBAP2) preserves the expressions of ZO-1 and occludin. (a) The expression of circUBAP2 in PMVECs treated with nonpulsatile or pulsatile flow for 6 hours; (b) the expressions of ZO-1 and occludin in PMVECs treated with circUBAP2 knockdown or scrambled siRNA using western blot assay; (c) immunofluorescent analysis of the ZO-1 distribution in PMVECs treated with circUBAP2 silencing or scramble siRNA; (d) immunofluorescent analysis of the occludin distribution in PMVECs treated with circUBAP2 silencing or scrambled siRNA. ^∗∗^Significantly different between groups, *p* < 0.01. Abbreviations: ZO-1: zonula occludens-1; DAPI: 4′,6-diamidino-2-phenylindole; PMVEC: pulmonary microvascular endothelial cell.

Consistent with the findings in animal models, hsa_circ_0007367 upregulation was also seen in PMVECs after 6-hour exposure to pulsatile flow (Figure 4(a)). To investigate the effects of hsa_circ_0007367 upregulation on endothelial integrity, we silenced hsa_circ_0007367 in PMVECs using siRNAs. Western blot assay revealed downregulation of ZO-1 and occludin in PMVECs with hsa_circ_0007367 knockdown, as compared to the scrambled control (Figure 4(b)). Meanwhile, the distribution of ZO-1 and occludin became detached when silencing hsa_circ_0007367 as compared to the control siRNA (Figures 4(c) and 4(d)).

### 3.4. hsa_circ_0007367 (UBAP2) Is Required for the Upregulation of ZO-1 and Occludin Induced by Pulsatile Flow

**Figure 5:**
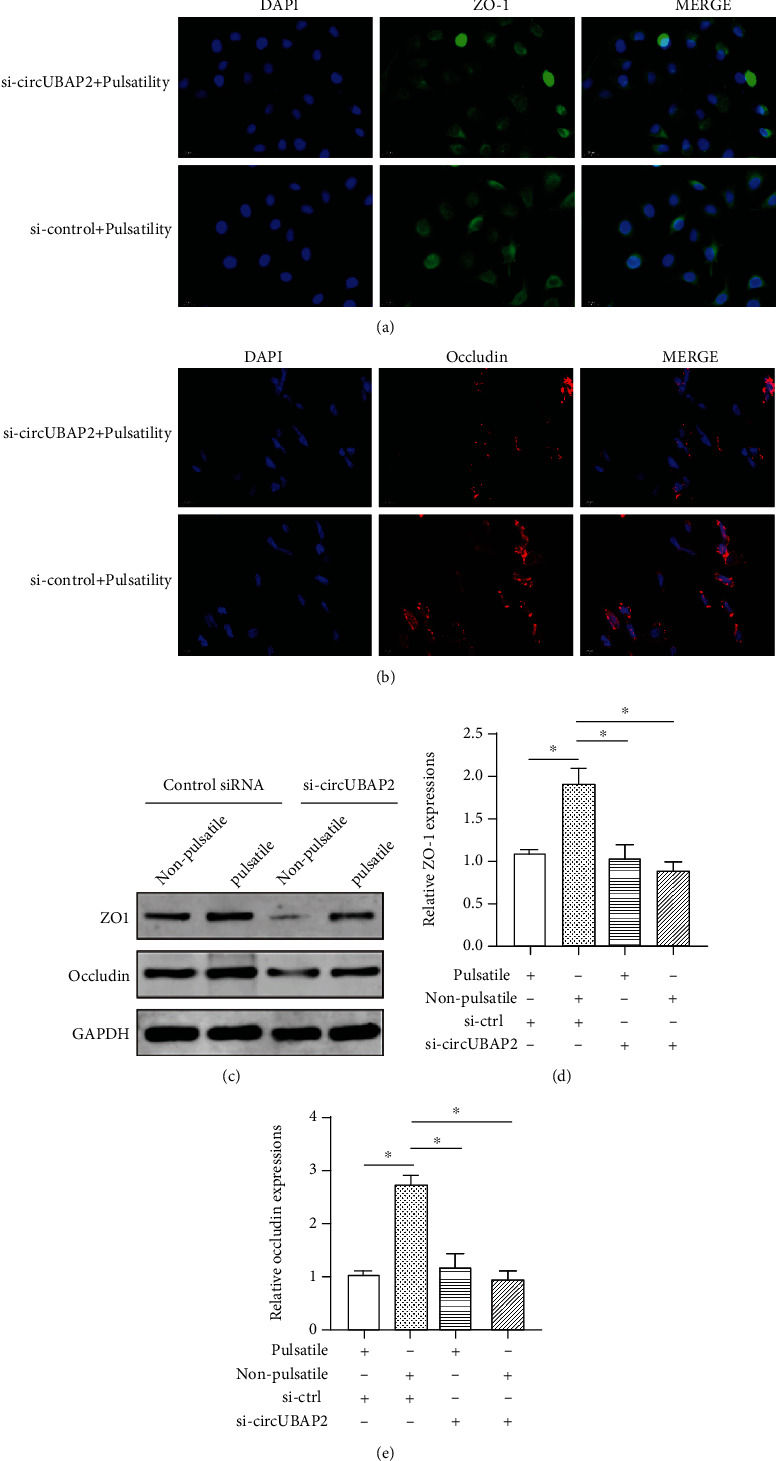
hsa_circ_0007367 (UBAP2) is needed for the pulsatility-mediated ZO-1 and occludin upregulation. (a) The distribution of ZO-1 in PMVECs treated with control siRNA or siRNA targeting circUBAP2, followed by 6-hour exposure to pulsatile flow; (b) the distribution of occludin in PMVECs treated with control siRNA or siRNA targeting circUBAP2, followed by 6-hour exposure to pulsatile flow; (c) the expressions of ZO-1 and occludin detected with western blotting in PMVECs treated with control siRNA or si-circUBAP2, followed by 6-hour exposure to pulsatile flow or nonpulsatile flow; (d) the expression of ZO-1 mRNA in PMVECs treated with control siRNA or si-circUBAP2, followed by 6-hour exposure to different flow patterns; (e) the expression of occludin mRNA in PMVECs treated with control siRNA or si-circUBAP2, followed by 6-hour exposure to different flow patterns. ^∗^*p* < 0.05; ^∗∗^*p* < 0.01. Abbreviations: ZO-1: zonula occludens-1; DAPI: 4′,6-diamidino-2-phenylindole; si: small interfering RNA; Ctrl: control; PMVEC: pulmonary microvascular endothelial cell.

To investigate if hsa_circ_0007367 is necessary for the pulsatility-mediated upregulation of tight junction biomarkers, PMVECs were transfected with scrambled siRNA or siRNA targeting hsa_circ_0007367, followed by 6-hour exposure to nonpulsatile flow or pulsatile flow. After silencing the expression of hsa_circ_0007367, the distribution of ZO-1 and occludin became abnormal in PMVECs exposed to pulsatile flow (Figures 5(a) and 5(b)). Consistent with the findings of immunofluorescent analyses, upregulation of ZO-1 and occludin mediated by pulsatile flow was abolished by knockdown of hsa_circ_0007367. PMVECs that were treated with control siRNA and were exposed to pulsatility had the highest ZO-1 and occludin expressions (Figures 5(c)–5(e)). These results suggest that hsa_circ_0007367 is an essential element for the upregulation of ZO-1 and occludin induced by pulsatility.

### 3.5. Pulsatility Suppresses Adhesion Molecules and Endothelial Inflammatory Pathways in a hsa_circ_0007367-Dependent Manner

**Figure 6:**
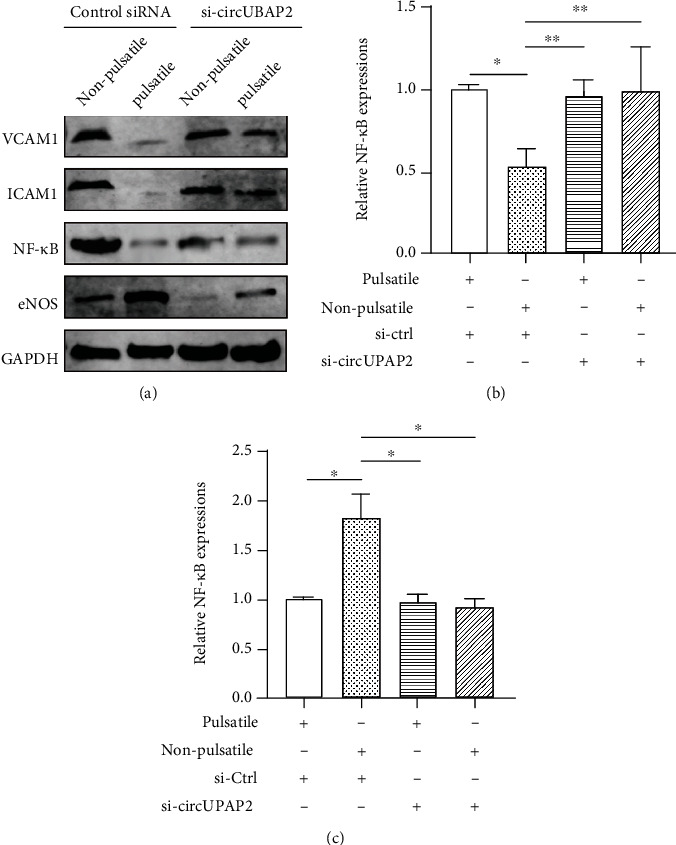
hsa_circ_0007367 (UBAP2) is required for the suppression of endothelial adhesion and inflammation mediated by pulsatile flow. (a) The expressions of endothelial adhesive molecules, NF-*κ*B, and eNOS proteins using western blotting in PMVECs treated with control siRNA or si-circUBAP2, followed by 6-hour exposure to pulsatile flow or nonpulsatile flow; (b) the expression of NF-*κ*B mRNA in PMVECs treated with different siRNAs and different flow patterns; (c) the expression of eNOS mRNA in PMVECs treated with different siRNAs and different flow patterns. ^∗^*p* < 0.05; ^∗∗^*p* < 0.01. Abbreviations: ZO-1: zonula occludens-1; DAPI: 4′,6-diamidino-2-phenylindole; si: small interfering RNA; Ctrl: control; eNOS: endothelial nitric oxide synthases; PMVEC: pulmonary microvascular endothelial cell.

There is a possible interaction between microcirculation and inflammation. To examine how pulsatility attenuates endothelial inflammation, the expressions of adhesion molecules and inflammatory pathways were measured (Figures 6(a)–6(c)). The pulsatile flow significantly reduced the levels of adhesion molecules including VCAM-1 and ICAM-1, while the reduction could be abolished by hsa_circ_0007367 silencing. The NF-*κ*B, a known inflammatory molecule, was downregulated under pulsatile flow on the premise of hsa_circ_0007367 expression. Similarly, pulsatility exhibited downregulation of eNOS, which could be reversed by hsa_circ_0007367 silencing as well. These results suggest that pulsatile flow suppresses endothelial adhesion and inflammation in a hsa_circ_0007367-dependent fashion.

## 4. Discussion

We present a pulsatile modification of the flow pattern, which offers a feasible approach to improve microcirculatory perfusion and stabilize endothelial integrity during ECMO. In particular, this protective effect is dependent on the expression of hsa_circ_0007367 (UBAP2). We demonstrate that pulsatile flow upregulates the expression of hsa_circ_0007367, leading to the upregulation of endothelial tight junction markers ZO-1 and occludin, followed by modulating the eNOS activity, endothelial adhesion, and the NF-*κ*B pathway.

It has been well recognized that ECMO rescues macro-hemodynamics promptly; however, recovery of the impaired microcirculation is not guaranteed in critically ill patients. The dearth of microcirculatory coherence, leading to the inability of macro-hemodynamics to resuscitate microcirculation, had been characterized in cardiogenic shock [12, 13] and sepsis [14]. Recent studies have proved that endothelial integrity breakdown with subsequent inflammation is the crux of microcirculatory malperfusion [15]. Furthermore, active cardiovascular disorders like postoperative low cardiac output or cardiogenic shock may trigger cytokine storm which further complicates the situation. Hence, preserving microcirculatory function and endothelial integrity is essential for ECMO patients.

The nonpulsatile flow is relevant to diminished shear stress and decreased production of nitric oxide during cardiopulmonary bypass [16], while pulsatile flow offers biomimetic pulsatility on endothelial cells, therefore alleviating endothelial inflammatory response [17, 18]. Preservation of pulsatility also improves microcirculatory perfusion during cardiopulmonary bypass and throughout the perioperative course [19]. We previously confirmed the protective effects of pulsatility on ECMO, which generates more hemodynamic energy, reduces proinflammatory cytokines [7], and inhibits endothelial-to-mesenchymal transformation in endothelial cells as compared with the conventional ECMO [8]. In this study, we further confirmed that pulsatility benefits microcirculatory perfusion and endothelial integrity during ECMO support. It is noteworthy that pulsatile ECMO is a subtype of V-A ECMO but not veno-venous (V-V) ECMO, in which the oxygenated blood is returned to the venous system.

Impaired endothelial integrity is a secondary event of microcirculatory malperfusion. When blood flow is abnormally disturbed, the distribution of wall shear stress on endothelial cells becomes irregular [8, 20], subsequently resulting in the endothelial barrier impairment. Vascular endothelial cells are jointed together via intercellular junctions, which will be disturbed when the endothelial barrier is disrupted [21]. Focusing on ZO-1 and occludin, two tight junction biomarkers, we observed that these molecules were ill arranged and discontinuously distributed in PMVECs under nonpulsatile flow. After pulsatile modification of the flow pattern, the arrangement of tight junctional molecules looks more well organized.

Compared to the nonpulsatile flow, the pulsatile flow may offer mechanical force which is mechanically transducted into endothelial cells, followed by the mechanical-to-biological transformation with changes in downstream signaling [22]. We provide evidence confirming that pulsatile flow upregulates the expression of hsa_circ_0007367, a microcirculation highly related circRNA that positively regulates ZO-1 and occludin. Moreover, we could demonstrate decreased expressions of endothelial adhesive molecules and the NF-*κ*B signaling. These suppressive effects on inflammation are hsa_circ_0007367 dependent. Although immune-modulating effects of pulsatility were confirmed previously [7], whether these anti-inflammatory actions are the direct impacts of hsa_circ_0007367 on immune cells or the secondary effects of microcirculatory improvement is still unclear. We believed that pulsatility brings about immunomodulatory effects on the inflammatory microenvironment under ECMO.

The ubiquitin-associated protein 2 (UBAP2) gene has a domain for ubiquitination, which functions diversely in various biological processes, such as metabolism, cell apoptosis, transcription, and inflammation responses [23, 24]. Until recently, the biological function of the UBAP2 gene has been studied mainly in cancers; UBAP2 is associated with the metastasis of prostate carcinoma [25], while conversely, UBAP2 is related to better prognosis in hepatic cellular carcinoma [26]. Originating from the host gene UBAP2, hsa_circ_0007367 is cyclized between the 11 and 13 exons, with a sliced length of 472 nt, while its biological function is rarely documented. We herein, for the first time, demonstrate that hsa_circ_0007367 highly correlates with microcirculation and regulates endothelial integrity during ECMO.

We are starkly aware of the limitations of this work. Firstly, we are still not able to conclude how microcirculation communicates with inflammation before completely studying the effects of hsa_circ_0007367 and pulsatility on immune cells. Secondly, the duration of the experimental ECMO is relatively short. Patients with cardiopulmonary failure usually receive ECMO support for weeks or even months. Thirdly, the pulsatile flow was established through the carotid artery cannulation in an antegrade manner. The ECMO flow, however, is usually pumped retrograde through the femoral artery and might compete with blood flow from the heart. It is still unclear how sites of cannulation affect ECMO pulsatility. Finally, given the relatively small body weight of the beagles to mimic the ECMO pathophysiology in pediatric patients, our results need further validation in larger animal models with longer ECMO duration. Our findings, however, strengthen the evidence for the benefits of pulsatility during ECMO support.

## 5. Conclusion

In conclusion, the pulsatile modification in ECMO enhances microcirculatory perfusion and stabilizes the endothelial integrity by upregulating the expression of hsa_circ_0007367 (UBAP2), which exerts protective effects in microcirculation and attenuates endothelial inflammation. Future studies are warranted to refresh these results.

## Figures and Tables

**Table 1:** Primers for qRT-PCR.

Genes	Primer sequences
hsa_circ_0007367	Forward 5′-TCCTCAGTCATCTTGCTTTCTG-3′ Reverse 5′-TGAGGAACAGGCTTCTGGAG-3′
ZO-1	Forward 5′-ACCCGAAACTGATGCTGTGGATAG-3′ Reverse 5′-AAATGGCCGGGCAGAACTTGTGTA-3′
Occludin	Forward 5′-ACGGACCCTGACCACTATGA-3′ Reverse 5′-TCAGCAGCAGCCATGTACTC-3′
NF-*κ*B	Forward 5′-CTGATGGCACAGGACGAGAA-3′ Reverse 5′-TGGGCTATCTGCTCAATGACAC-3′
eNOS	Forward 5′-TCCAGAGCATACCCGCACTTC-3′ Reverse 5′-GTCCAGACGCACCAGGATTG-3′
GADPH	Forward 5′-GAAGGTGAAGGTCGGAGTCAAC-3′ Reverse 5′-CATCGCCCCACTTGATTTTGGA-3′

**Table 2:** Macrocirculatory parameters and blood gas during ECMO support.

Time points	Groups	MAP (mmHg)	MND (*μ*g/kg/min)	pH	PaO_2_ (mmHg)	Hb (g/L)	SaO_2_ (%)	Lac (mmol/L)
Baseline (*T*_0_)	NP-ECMO	60.20 ± 7.19	0.15 ± 0.09	7.26 ± 0.07	79.00 ± 16.05	96.00 ± 9.38	95.40 ± 1.95	4.12 ± 1.02
	P-ECMO	61.00 ± 9.11	0.17 ± 0.10	7.26 ± 0.05	88.40 ± 11.04	102.00 ± 5.45	96.80 ± 1.92	4.66 ± 0.72
ECMO 1 h (*T*_1_)	NP-ECMO	62.60 ± 9.81	0.08 ± 0.06	7.30 ± 0.05	187.60 ± 20.07	91.00 ± 5.92	99.60 ± 0.55	2.12 ± 0.40
	P-ECMO	59.40 ± 8.93	0.06 ± 0.04	7.35 ± 0.06	217.20 ± 44.28	84.00 ± 7.18	99.80 ± 0.45	2.56 ± 0.70
ECMO 2 h (*T*_2_)	NP-ECMO	61.20 ± 5.36	0.09 ± 0.05	7.40 ± 0.04	188.60 ± 20.55	85.60 ± 5.13	99.80 ± 0.45	1.60 ± 0.25
	P-ECMO	62.40 ± 7.60	0.07 ± 0.08	7.42 ± 0.05	207.80 ± 24.08	81.00 ± 5.29	99.60 ± 0.55	1.88 ± 0.24
ECMO 4 h (*T*_3_)	NP-ECMO	63.00 ± 8.15	0.07 ± 0.03	7.40 ± 0.03	195.00 ± 27.38	82.20 ± 2.86	99.80 ± 0.45	1.16 ± 0.17
	P-ECMO	61.00 ± 9.54	0.05 ± 0.08	7.38 ± 0.04	205.80 ± 20.19	78.20 ± 4.49	99.20 ± 0.84	1.22 ± 0.19
ECMO 6 h (*T*_4_)	NP-ECMO	62.00 ± 6.32	0.05 ± 0.05	7.40 ± 0.03	186.40 ± 11.37	82.40 ± 2.88	99.60 ± 0.55	0.94 ± 0.15
	P-ECMO	61.80 ± 6.61	0.06 ± 0.08	7.39 ± 0.03	188.40 ± 19.77	77.80 ± 4.27	99.80 ± 0.45	1.12 ± 0.33

Abbreviations: ECMO: extracorporeal membrane oxygenation; MAP: mean arterial pressure; MND: mean noradrenaline dosage; PaO_2_: arterial partial pressure of oxygen; Hb: hemoglobin; SaO_2_: arterial oxygen saturation; Lac: lactate.

## Data Availability

We would like to declare the data availability issue for this manuscript. The data used to support the findings of this study are available from the corresponding author upon reasonable request.
